# Bioanalytical Method Using Ultra-High-Performance Liquid Chromatography Coupled with High-Resolution Mass Spectrometry (UHPL-CHRMS) for the Detection of Metformin in Human Plasma

**DOI:** 10.3390/molecules25204625

**Published:** 2020-10-11

**Authors:** Ye-Ji Kang, Hyeon-Cheol Jeong, Tae-Eun Kim, Kwang-Hee Shin

**Affiliations:** 1 Research Institute of Pharmaceutical Sciences, College of Pharmacy, Kyungpook National University, Daegu 41566, Korea; yeyezzy@knu.ac.kr (Y.-J.K.); houkiboshi01@knu.ac.kr (H.-C.J.); 2 Department of Clinical Pharmacology, Konkuk University Medical Center, Seoul 05029, Korea; tekim@kuh.ac.kr

**Keywords:** metformin, bioanalytical method, ultra-high-performance liquid chromatography (UHPLC), high-resolution mass spectrometry (HRMS), HILIC

## Abstract

Metformin is the first-line medicine for the treatment of type 2 diabetes. Drug interactions between metformin and other drugs, food, or beverages cannot only cause changes in the pharmacokinetic profiles but also affect the efficacy of metformin. The purpose of this study was to develop a rapid and reliable bioanalytical method for the detection of plasma metformin concentration in humans. To remove interfering substances in plasma, acidified acetonitrile (acetonitrile containing 0.1% formic acid) was added to samples. Ultra-high-performance liquid chromatography (UHPLC) coupled with high resolution mass spectrometry (HRMS) was used to analyze metformin and its internal standard (metformin-d6). Analyte separation was performed on a BEH HILIC analytical column (100 × 2.1 mm, 1.7 μm) using a gradient elution of 0.1% formic acid (A) and acetonitrile with 0.1% formic acid (B). The total chromatographic run time was 2 min. The developed method was validated for its linearity, accuracy and precision, selectivity (signal of interfering substance; analyte, lower limit of quantification (LLOQ) ≤ 20%; IS, IS ≤ 5%), sensitivity (LLOQ, 5 ng/mL; S/N ratio ≥ 10), stability (low quality control (LQC, 15 ng/mL), 2.95–14.19%; high quality control (HQC, 1600 ng/mL), −9.49–15.10%), dilution integrity (diluted QC (4000 ng/mL); 10-folds diluted QC (400 ng/mL); 5-folds diluted QC (800 ng/mL); accuracy, 81.30–91.98%; precision, ≤4.47%), carry-over (signal of double blank; analyte, LLOQ ≤20%; IS, IS ≤5%), and matrix effect (LQC, 10.109%; HQC, 12.271%) under various conditions. The constructed calibration curves were shown linear in the concentration range of 5–2000 ng/mL, with within- and between-run precision values of <8.19% and accuracy in the range of 91.13–105.25%. The plasma metformin concentration of 16 healthy subjects was successfully measured by applying the validated bioanalytical method.

## 1. Introduction

Type 2 diabetes mellitus (T2DM) is one of the major concerns of public health around the world, and it currently represents major medical and social problems due to high rates of disability and mortality [1,2]. T2DM is often caused by a gradual decrease in β-cell insulin secretion due to insulin resistance [3]. Compared to other drugs indicated to T2DM, such as sulfonylurea and glinide, metformin is mainly used as a first choice drug due to its great safety and relatively low risk of side effects, such as hypoglycemia and weight gain. Physiologically, metformin has been shown to decrease hepatic glucose production on the liver, and acts to increase glucose utilization on the gut. At the molecular level, this medication suppresses the mitochondrial respiratory chain in the liver, enhancing insulin sensitivity [4]. When oral metformin is absorbed into the gastrointestinal tract, it is a substrate drug for drug carriers such as organic cation transporter (OCT1) and multidrug and toxin extrusion protein (MATE1/2) kidney transport. If administered with inhibitors or inducers of these transporters, changes in blood concentration and an effect on blood sugar are expected. Therefore, advanced bioanalytical methods are needed to quantify the plasma metformin concentration in humans.

Various bioanalytical methods for the determination of metformin in human-derived materials have been developed, including gas chromatography (GC) with mass spectrometry [5] and liquid chromatography (LC) with UV detection [6,7] or mass spectrometry [8,9,10]. GC methods require a complex derivatization procedure before mass spectrometry to compensate chromatographic issues as low volatility [5]. The reported LC methods with UV detection have shown higher lower limit of quantification (LLOQ) concentration levels [6,7]. Additionally, LC-tandem mass spectrometry suffers from several disadvantages, such as requiring long chromatographic times [8,9,10], a lack of sensitivity [9,10,11], and the use of complicated sample preparation procedures, including liquid-liquid extraction [9].

The high-resolution mass spectrometry (HRMS) has excellent performance in targeted and nontargeted screening and characteristic analysis, but there are studies on the quantification of small analytes [12,13,14]. The HRMS used in this study is a recent hybrid orbitrap mass spectrometer, which combines the orbitrap of high-resolution performance and the quadrupole of high selectivity. Thus, an excellent result can be obtained with sensitivity, selectivity, accuracy, and precision for quantitative analysis using full scan reaction mode and parallel reaction monitoring (PRM).

To the best of our knowledge, no analysis method for plasma metformin determination using UHPLC-HRMS has been reported, to the best of our knowledge. The goal of this study was to develop a rapid and reliable bioanalytical method for determining the plasma metformin concentration in humans.

## 2. Results

### 2.1. Method Development

**Figure 1:**
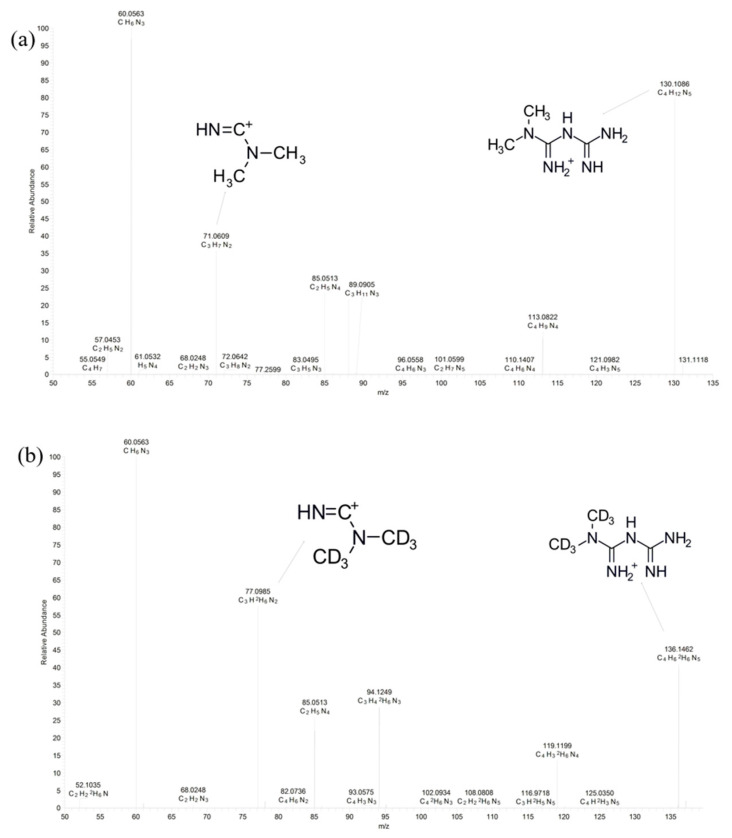
Mass spectrum and chemical structures of (**a**) metformin and (**b**) deuterated internal standard (metformin-d6) in electrospray ionization (ESI) positive mode.

The mass spectrum of metformin and the internal standard (IS; metformin-d6) are presented with chemical structures in Figure 1. Method development considered molecular properties, such as chemical formula (molecular structure), molecular weight (129.164 g/mol), pKa (11.5–12.4), water solubility (logP = −0.27), and the polarity of metformin [15]. Metformin and the IS, which both contain alkylamine groups in their structures, are strongly basic compounds and could be detected under positive ESI conditions; as a result, metformin and the IS showed good responses. To determine a suitable protein precipitation method, various solvents, such as acetonitrile and methanol, were used. Acetonitrile with 0.1% formic acid had the best precipitation efficiency compared to the other organic solvents tested. The metformin had a polarizing property in nature, so a slightly acidic solution was more suitable than just acetonitrile.

To set elution conditions, various mobile solvents such as deionized water or 0.1% formic acid and acetonitrile or acetonitrile containing 0.1% formic acid were tested for separation using isocratic and gradient methods. The results showed that gradient elution was more appropriate than isocratic elution. Because the acidic modifier in the mobile phase could improve the peak shape and the sensitivity of the assay, a mobile phase consisting of 0.1% formic acid (**A**) and acetonitrile containing 0.1% formic acid (**B**) was used.

As metformin is a small molecule, when analyzing biological samples, there was a problem with the analysis in which the matrix effect was generated. Therefore, in this study, a mass exclusion list of MS conditions was used to disregard interfering ions existed in the background and was useful for increasing the peak sensitivity of the analyte. This exclusion list was filled with interfering ions extracted from the blank sample.

### 2.2. Linearity and Sensitivity

**Figure 2:**
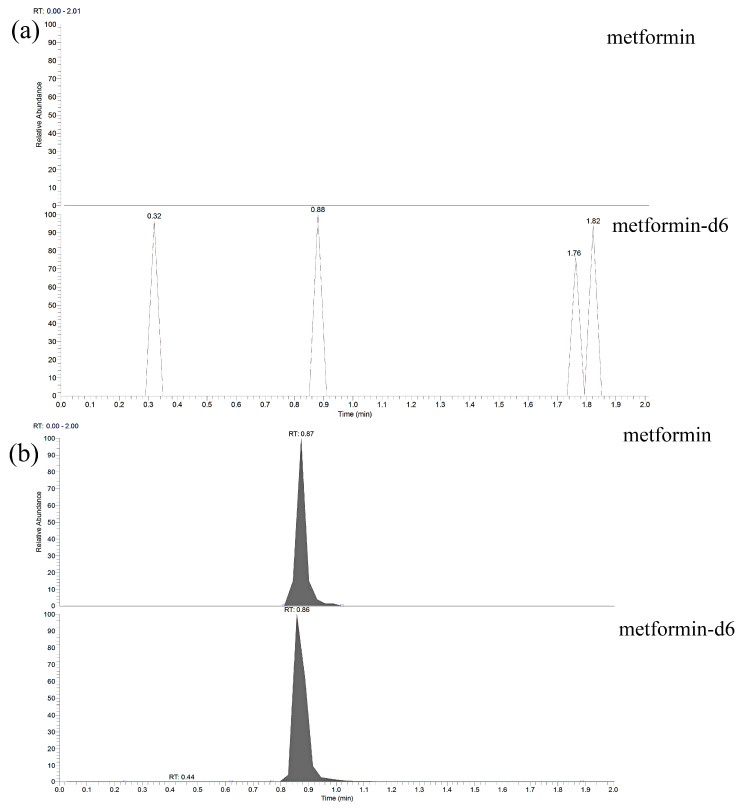
Representative chromatograms of (**a**) double-blank human plasma and (**b**) the lower limit of quantification samples.

The linearity for plasma metformin evaluated in the concentration range of 5–2000 ng/mL, and linear regression with the equation y = ax + b was produced by applying a weight of 1/x to the peak area ratio of the analyte to the IS obtained from the calibration curve. According to the calculation of the deviation of the measured and theoretical values for the eight standard concentrations used in the preparation of the calibration curve, more than 75% of the standard concentrations were based only on the criteria (within 15% and within 20% of LLOQ). This method was satisfied with the criteria, and the correlation coefficient (r) was > 0.998. The verifications about linearity are summarized in Table 1. Metformin in plasma maintains a sensitivity at which the S/N ratio was more than 10, comparing the responses of the analyte at the LLOQ the double-blank plasma in which neither metformin nor IS was spiked. Chromatogram of double-blank and the LLOQ samples are shown in Figure 2.

### 2.3. Accuracy and Precision

As a result of the evaluation of accuracy and precision for the four concentrations (lower limit of quantification; LLOQ, low quality control; LQC, middle quality control; MQC, and high quality control; HQC), the accuracy was 91.13–105.25%, and the within- and between-run precision values were within 8.19%. The results are arranged in Table 2.

### 2.4. Selectivity and Matrix Effects

As a result of analyzing the selectivity, the signal of the interfering substance met the criteria below 20% of the LLOQ of the analyte and below 5% of the IS during peak retention times in the chromatogram of LLOQ. The mean matrix effects for metformin in human plasma ranged from 85.7–113.2% with the corresponding CV (%) values of 15% or less at the LQC and HQC. The results of selectivity and matrix effects are shown in Table 3.

### 2.5. Stability and Recovery

The stability of the stock and working solutions were conducted on varied conditions, and the results are arranged in Table 4. Both the short-term working solution and stock solution had stability at room temperatures for 6 h. The LQC (15 ng/mL) and HQC (1600 ng/mL) samples that were measured after three freeze-thaw cycles were within 15% of the concentration change. The re-injection concentration change was within 15%. The processed sample stability (PSS) sample was stable for 24 h in the autosampler at 10 °C. The results showed that the developed method was stable under the conditions evaluated. The results of the recovery were within 15% of the precision at each concentration (LQC, MQC, and HQC) and within 15% of the %CV. However, the stability of metformin in plasma at room temperature for 6 h was marginally acceptable with 15.10% of CV.

### 2.6. Application of the Method in Human Plasma Samples

**Figure 3:**
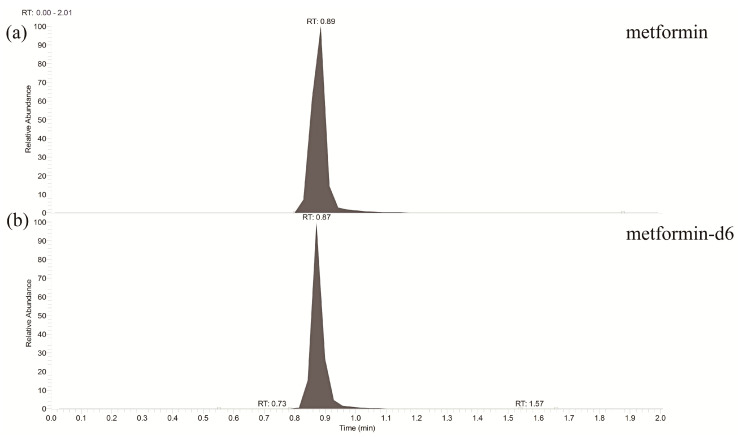
Representative chromatograms of (**a**) metformin and (**b**) internal standard in plasma after oral administration of 1000 mg of metformin.

**Figure 4:**
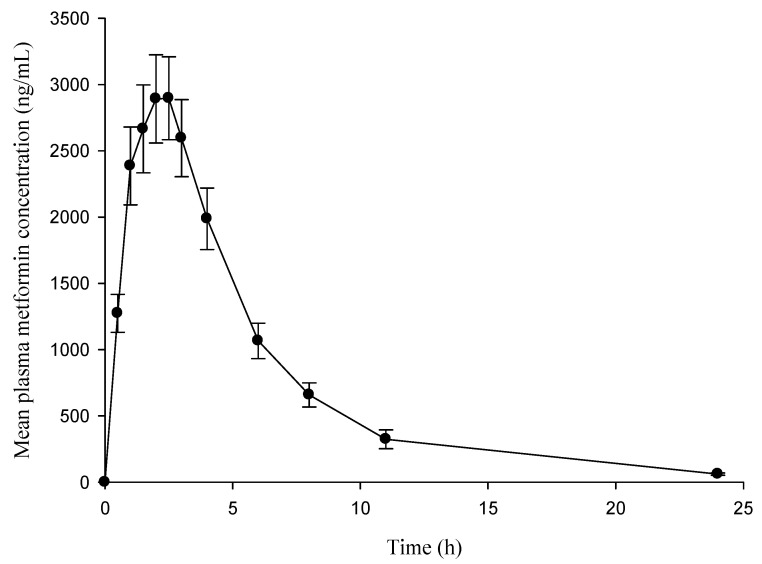
Mean time-concentration profile after a single 1000 mg dose of metformin. Error bars represent the standard errors (SEs).

The validated method was applied to the determination of plasma metformin in healthy subjects, successfully. Chromatogram of metformin and IS in plasma after oral administration of 1000 mg of metformin are shown in Figure 3. Linearity and reproducibility were evaluated for each analysis batch. The plasma metformin concentration-time profiles and pharmacokinetic parameters are presented in Figure 4 and Table 5, respectively.

## 3. Discussion

To measure the plasma concentration of metformin, an analytical method using UHPLC-HRMS was developed and validated. Separation on a BEH HILIC column using the mobile phase containing 0.1% formic acid in both LC/MS grade water and acetonitrile showed the best peak shape, lowest background noise level, and best reproducibility compared to the other mobile phases tested. The mass detector conditions were optimized using ESI positive mode for metformin and the IS. The developed analytical method showed adequate linearity (r ≥ 0.993) for the concentration range of 5–2000 ng/mL and suitable accuracy (91.13–105.25%) and precision (%CV within 8.19%) in all analysis batches. The stability of metformin under varied conditions at two concentrations (LQC and HQC) was within a 15% change. It has been confirmed that the analyte is stable in the plasma during storage conditions and analysis processes. The plasma metformin concentration in 16 healthy subjects was successfully determined using the developed method. This study improved various analysis conditions, such as the lower LLOQ concentration (5 ng/mL), reduced chromatographic time (2 min), and a simple sample preparation procedure (protein precipitation) compared with previous studies.

Improved performance was obtained in this study using HRMS combined with a HILIC analytical column. In some previous studies, the LLOQ concentration was greater than 5 ng/mL [6,9,10,11,16]. In the Fachi et al. paper, when ultra-performance liquid chromatography quadrupole time-of-flight mass spectrometry (UPLC-QToF-MS) was used to develop an analysis method for quantifying various drugs including metformin in human plasma, the concentration of LLOQ was 250 ng/mL [16]. UPLC-QToF-MS is also HRMS, but in this study, the LLOQ concentration was 5 ng/mL and was determined using HRMS combined with a HILIC analytical column. The Q-Exactive used in this study is a recent hybrid Orbitrap mass spectrometer that combined an orbitrap and a quadrupole [17]. Hu et al.’s study referred to this instrument as having slightly better performance with resolving power up to 70,000 full width at half maximum (FWHM) at *m*/*z* 200 and a mass shift that referred to mass accuracy less than 2 ppm. In addition, several HRMS parameters, such as the automatic gain control (AGC) target, maximum injection time (IT), lock mass, and mass tolerance window (MTW), have been regulated to optimize quantitative analysis [18]. These also contribute to achieving high sensitivity and selectivity. This study set the MS conditions by mass tuning using metformin and the IS standard solution with a concentration of 10 ppm. The AGC target, maximum IT, and MTW were set at 5 × 10^4^ (arbitrary units), 100 ms, and 3.0 ppm, respectively, in this study. Moreover, to elute polar compounds such as metformin, the HILIC analytical column offers improved sensitivity because the percentage of organic solvents in the mobile phase is high [17]. In previous metformin quantitative analysis studies [6,8,11,19,20], an isocratic method was generally used, but in this paper, a gradient method was used to shorten the time of peak elution. Therefore, the time was shortened by 1.5 min compared with the 3.5 min in the Mistri et al. study, which was the longest time of previous studies.

The sample preparation performed protein precipitation using acetonitrile with 0.1% formic acid. The sensitivity and peak shape improved when using acetonitrile with 0.1% formic acid than acetonitrile. Compared with previous studies using liquid–liquid extraction or solid-phase extraction [9], protein precipitation is a rapid and simple preparation method. Last, the total chromatographic run time in this study was 2 min. In general, the analysis time has been longer than 2 min in some published studies [8,9,10].

Among previous studies of quantitative analysis for metformin in human plasma, the LLOQ values were ranged from 1 ng/mL to 50 ng/mL [6,8,9,11,21]. Of these, there was a study that used LC-MS/MS with a LLOQ concentration of 1 ng/mL, and the S/N ratio was 20 [8]. Shah et al. showed that the calibration curves were linear in the concentration range of 1–1000 ng/mL with an r^2^ ≥ 0.9978, and the accuracy and precision values ranged from 96.2–103.3% and 1.06–4.31%, respectively. In the Shah et al. study, the conditions were, in general, well-identified using a HILIC column and changing the buffer concentration, nature, and proportion of the organic diluents, pH, and temperature. In the Shah et al. study, the total chromatographic run time was 4 min, while in this study, although the LLOQ concentration was higher, the analysis time was shortened to 2 min by changing the conditions of mobile phases A and B by adding 0.1% formic acid. In other study, the analytical method was linear within the range of 2–2000 ng/mL, with a total run time of 3.4 min [21]. Instead of acetonitrile used as protein precipitation in the Wang et al. study, our study had a high efficiency of sedimentation using acetonitrile with 0.1% formic acid. Thus, comparing precision values of <8.60% and accuracy ranges of 94.1–106.9% in the Wang et al. study, the precision (<8.19%) and accuracy (91.1–105.2%) were excellent in the current study.

This study represented that the UHPLC-HRMS can be used in the detection and quantitative analysis of plasma metformin in humans. The developed analytical method showed good linearity, sensitivity, accuracy and precision, and repeatability. This analytical method with UHPLC-HRMS could be applied for pharmacokinetic studies of various drugs.

## 4. Materials and Methods

### 4.1. Chemicals, Reagents, and Materials

Metformin hydrochloride, a standard reference material, was obtained from Sigma-Aldrich (St. Louis, MO, USA) with a purity of 99.9% (pharmaceutical grade). Metformin-d6 as an internal standard (IS) was purchased from Toronto Research Chemicals (Toronto, ON, Canada) and had a purity of 97% (pharmaceutical grade). LC-MS-grade acetonitrile and deionized water were purchased from Merck (Darmstadt, Germany). ACS grade formic acid was purchased from Sigma-Aldrich. Whole blood was collected in a tube containing EDTA-K3 from healthy volunteers at Konkuk University Medical Center (Seoul, Korea) and stored at −80 °C until analysis.

### 4.2. Liquid Chromatography and Mass Spectrometric Conditions

The UHPLC system, an Ultimate 3000 (Thermo Fisher Scientific, CA, USA), consisted of a binary pump, an autosampler, and a BEH HILIC 100 × 2.1 mm, 1.7 μm analytical column (Waters, MA, USA) maintained at 45 °C. For mobile phase, 0.1% formic acid (**A**) and acetonitrile of 0.1% formic acid (**B**) was used. The flow rate was maintained at 0.4 mL/min by applying gradient elution as follows: 40% **B**, ramp to 90% **B** from 0–0.5 min, hold at 90% **B** until 0.9 min, return to 40% **B** from 0.9–1.1 min, and hold at 40% **A** until 2 min; the total run time was 2 min. The vials containing the samples existed in the autosampler set to 10 °C, and 5μL was injected into the instrument. Detection of metformin and the IS was performed on a Q Exactive™ Focus Hybrid Quadrupole-Orbitrap™ Mass Spectrometer (Thermo Fisher Scientific) with built-in heated electrospray ionization (HESI) in positive ion mode. Scan was performed using parallel reaction monitoring (PRM) mode at a resolution of 7000, and the ion mass transitions for metformin (m/z 130.1087 → 71.0609) and the IS (m/z 136.1462 → 77.0985) were present. The optimized source-dependent parameters were sheath gas flow rate: 10 (arbitrary units); aux gas flow rate: 0 (arbitrary units); spray voltage: 2.5 kV; capillary temperature: 320 °C; and normalized collision energy (NCE) 40 for metformin and 50 for IS. Thermo Scientific Xcalibur (ver. 4.1) (Thermo Fisher Scientific) was used for UHPLC-HRMS system control and data processing.

### 4.3. Standard and Quality Control Preparation

The metformin and IS stock solutions were constructed at a concentration of 1 mg/mL using 100% methanol. The stock solution of metformin was further diluted with the same solvent to obtain working standard solutions at the concentrations of 50, 100, 200, 400, 1000, 4000, 10,000, and 20,000 ng/mL. Calibration standard samples were prepared from metformin working standard solutions spiked with blank plasma to final concentrations of 5, 10, 20, 40, 100, 400, 1000, and 2000 ng/mL. Quality control (QC) samples were prepared at concentrations of LQC (15 ng/mL), MQC (150 ng/mL), and HQC (1600 ng/mL) in the similar manner. The IS working solution was also prepared at a final concentration of 400 ng/mL using 100% methanol.

### 4.4. Sample Preparation

The frozen subject plasma samples were thawed at room temperature (25 °C). To a 100 μL plasma sample, 50 μL of IS solution (400 ng/mL) was added and mixed for 10 s. The mixtures were precipitated using 500 μL of 100% acetonitrile with 0.1% formic acid and centrifuged at 13,000 rpm, 4 °C for 5 min. The supernatant was transferred into vials, and 5 µL was injected into the chromatographic system (Thermo Fisher Scientific, CA, USA).

### 4.5. Bioanalytical Method Validation

The method was validated for linearity, accuracy and precision, sensitivity, stability, dilution integrity, recovery, carry-over, and matrix effects following the “Guidance for Industry Bioanalytical Method Validation” published by the USA Food and Drug Administration (USFDA 2018) [22] and the “Guideline on Bioanalytical Method Validation” for the Ministry of Food and Drug Safety in Korea (MFDS 2013) [23].

#### 4.5.1. Linearity and Sensitivity

A calibration curve was made from samples of working solutions and IS solutions in drug-free blank human plasma and included a double blank (no metformin or IS), blank (only IS), and eight standard calibration curve points in the concentration range from the LLOQ (5 ng/mL) to the upper limit of quantification (ULOQ, 2000 ng/mL). Sensitivity was assessed to calculate the S/N ratio by comparing the peak response of the double blank and LLOQ, and the criterion was an S/N ratio greater than 10 at the LLOQ.

#### 4.5.2. Accuracy and Precision

Accuracy and precision assessment were evaluated at the LLOQ, the LQC, MQC, and HQC. The test was repeated five times (within-run) and three times (between-run) in one batch for each concentration. The accuracy and precision had to be within ± 20% for the LLOQ and ± 15% for the LQC, MQC, and HQC.

#### 4.5.3. Selectivity and Matrix Effect

The selectivity evaluates the ability to quantitate correctly the concentration of the analyte when other substances coexist in the plasma. The matrix effect evaluates the effect of a substance in individual plasma on the accuracy and precision by affecting the reaction of analyte or IS. Samples were prepared in two concentrations (LQC and HQC) using six different individual plasma.

#### 4.5.4. Stability and Recovery

The stability was evaluated under various conditions, including short-term, short-term stock, freeze-thaw stability (FTS), re-injection, and processed sample stability (PSS). The stability tests of both short-term working solution and short-term stock solution stability were evaluated after the samples were kept at room temperature (25 °C) for 10 h. The FTS assessment was analyzed after three freeze–thaw cycles at storage and room temperature using the LQC and HQC samples. To measure the PSS, samples were stored in an autosampler at 10 °C overnight and analyzed. The recovery rate was assessed by comparing the detection reaction of the extracted samples with the detection reaction of the samples containing the analyte after applying the analyte to the plasma. The analysis of the analytes was conducted at each of the three concentrations (LQC, MQC, and HQC).

### 4.6. Application of the Method in Human Plasma Samples

The method was applied to analyze plasma metformin concentration obtained by 16 healthy subjects. To exclude other confounding factors of patients including co-medications, the study was conducted in healthy subject. The clinical study was conducted at Clinical Trial Center of Konkuk University Medical Center (Seoul, South Korea), and approval was obtained by the Institutional Review Board (IRB) of Konkuk University Medical Center (IRB No. KUH1280124). Whole blood was collected in tubes containing EDTA-K3, predose (0 h) and at 0.5, 1.0, 1.5, 2.0, 2.5, 3.0, 4.0, 6.0, 8.0, 11, and 24 h after oral administration of 1000 mg of metformin tablet. Collected whole blood was centrifuged at 3000 rpm for 10 min, then the separated plasma was stored at −80 °C until analysis. The concentration of metformin in the sample was determined by obtaining the peak area ratio of the analyte to the peak area of each IS obtained after UHPLC-HRMS analysis. In all analysis batches, the calibration curve was constructed over in the concentration range of 5–2000 ng/mL.

## 5. Conclusions

A simple, rapid, and reproducible UHPLC-HRMS method for the determination of metformin in human plasma was developed. This method satisfied all of the validation criteria recommend in the guidelines of bioanalytical method validation from the FDA and the MFDS, proving good linearity, sensitivity, accuracy and precision, and repeatability over the concentration range. The plasma metformin concentrations of 16 healthy subjects were successfully measured by applying the validated bioanalytical method.

## Figures and Tables

**Table 1:** Linearity data for assays metformin in human plasma.

Parameter	Value
Linearity range (ng/mL)	5–2000
Correlation coefficient	≥0.9980
Intercept ^a^	0.0005 ± 0.00002
Slope ^a^	0.0004 ± 0.0003
LLOQ (ng/mL)	5

^a^ Mean ± SD.

**Table 2:** Accuracy and precision data for assays metformin in human plasma (*n* = 5).

Parameter	LLOQ (5 ng/mL)	LQC (15 ng/mL)	MQC (150 ng/mL)	HQC (1600 ng/mL)
Within-run accuracy (%)	98.22	91.13	105.25	93.74
Between-run accuracy (%)	106.49	94.14	100.51	97.56
Within-run precision CV (%) ^a^	8.57	3.89	8.19	1.35
Between-run precision CV (%) ^a^	13.46	6.61	6.72	6.72

^a^ Coefficient of variation (%CV)

**Table 3:** The selectivity and matrix effects in human plasma (*n* = 6).

Number	Selectivity		Matrix Effect	
	Analyte	IS	LQC (15 ng/mL)	HQC (1600 ng/mL)
1	0	0.033	116.1	89.4
2	0	0	92.3	96.7
3	5.78	0	105.7	113.2
4	8.46	0	101.5	101.3
5	7.32	0.033	96.6	114.8
6	3.17	0	87.8	85.7
Mean ± SD	-	-	100.0 ± 10.1	100.0 ± 12.3
CV (%) ^a^	-	-	10.1	12.3

^a^ Coefficient of variation (%CV)

**Table 4:** Stability of metformin under various conditions at two concentrations (*n* = 3).

	LQC (15 ng/mL)	HQC (1600 ng/mL)
	% change	
Autosampler for 24 h	5.00	13.74
One freeze-thaw cycle	7.46	8.76
Three freeze-thaw cycles	2.95	−9.49
Plasma at room temperature for 6 h	13.33	15.10
Stock at room temperature for 6 h	14.19	3.03

**Table 5:** Pharmacokinetic parameters of metformin in human plasma after administration of a single 1000 mg dose of metformin.

Parameter	Value	
	Mean	SD
C_max_ (ng/mL)	3187.6	1316.2
T_max_ (h)	1.97	[1.00, 3.02] ^a^
AUC_last_ (ng·h/mL)	18474.6	8589.5

The peak plasma concentration (C_max_), the area under the plasma concentration-time curve from time zero to the time of the last measurable concentration (AUC_last_), and the time to reach the C_max_ (T_max_); ^a^ median [min, max].
